# Modulation of the COMT Val^158^Met polymorphism on resting-state EEG power

**DOI:** 10.3389/fnhum.2015.00136

**Published:** 2015-04-02

**Authors:** Silvia Solís-Ortiz, Elva Pérez-Luque, Mayra Gutiérrez-Muñoz

**Affiliations:** ^1^Laboratory of Cognitive Electrophysiology and Hormones, Department of Medical Sciences, University of GuanajuatoLeón, Mexico; ^2^Faculty of Psychology, Autonomous University of Nuevo LeónMonterrey, Mexico

**Keywords:** EEG, COMT, spectral power, dopamine, polymorphism

## Abstract

The catechol-O-methyltransferase (COMT) Val^158^Met polymorphism impacts cortical dopamine (DA) levels and may influence cortical electrical activity in the human brain. This study investigated whether COMT genotype influences resting-state electroencephalogram (EEG) power in the frontal, parietal and midline regions in healthy volunteers. EEG recordings were conducted in the resting-state in 13 postmenopausal healthy woman carriers of the Val/Val genotype and 11 with the Met/Met genotype. The resting EEG spectral absolute power in the frontal (F3, F4, F7, F8, FC3 and FC4), parietal (CP3, CP4, P3 and P4) and midline (Fz, FCz, Cz, CPz, Pz and Oz) was analyzed during the eyes-open and eyes-closed conditions. The frequency bands considered were the delta, theta, alpha1, alpha2, beta1 and beta2. EEG data of the Val/Val and Met/Met genotypes, brain regions and conditions were analyzed using a general linear model analysis. In the individuals with the Met/Met genotype, delta activity was increased in the eyes-closed condition, theta activity was increased in the eyes-closed and in the eyes-open conditions, and alpha1 band, alpha2 band and beta1band activity was increased in the eyes-closed condition. A significant interaction between COMT genotypes and spectral bands was observed. Met homozygote individuals exhibited more delta, theta and beta1 activity than individuals with the Val/Val genotype. No significant interaction between COMT genotypes and the resting-state EEG regional power and conditions were observed for the three brain regions studied. Our findings indicate that the COMT Val^158^Met polymorphism does not directly impact resting-state EEG regional power, but instead suggest that COMT genotype can modulate resting-state EEG spectral power in postmenopausal healthy women.

## Introduction

Catechol-O-methyl transferase (COMT) is the major mammalian enzyme involved in the metabolic degradation of released dopamine (DA) and accounts for more than 60% of DA degradation in the frontal cortex (Bertocci et al., 1991). The gene that encodes the COMT enzyme may influence cognition (Barnett et al., 2008; Solís-Ortiz et al., 2010) and brain function (Heinz and Smolka, 2006) through its effects on dopaminergic function. The human COMT gene contains a functional polymorphism in the coding sequence (a G to A substitution), resulting in a valine (Val) to methionine (Met) substitution at codon 158 (Val^158^Met), which affects the thermostability of the enzyme (Lachman et al., 1996). As a result of the allelic differences in enzymatic activity, Val carriers have less DA activity in the prefrontal cortex. The Met/Met polymorphism produces a less active enzyme, resulting in higher DA levels than in individuals with the Val/Val or the Val/Met polymorphism (Egan et al., 2001; Chen et al., 2004). The enzyme containing Met is unstable at 37°C and has one-third to one-fourth of the activity of the Val enzyme (Spielman and Weinshilboum, 1981; Lotta et al., 1995). The alleles are codominant, because heterozygous individuals have enzyme activity that is midway between homozygous individuals (Weinshilboum et al., 1999). Therefore, heterozygous phenotypes were not included in this study. The more robust statistical differences in COMT have been observed in heterozygous individuals with the Val/Val or Met/Met genotypes. Several experimental animal and human studies implicate COMT allelic variations in tuning cortical DA levels and consequent function (Diamond, 2007; Witte and Flöel, 2012). These studies indicate that this functional polymorphism accounts for most of the human variation in peripheral COMT activity. Therefore, an individual’s COMT genotype might also contribute to differences in prefrontal function (Akil et al., 2003).

In humans, COMT has gathered increasing interest in recent years with respect to the genetic disposition towards schizophrenia (Ehlis et al., 2007), anxiety traits (Lee and Prescott, 2014), depression (Shen et al., 2014), emotional disorders (Gohier et al., 2014) and deficits in cognitive prefrontal functions (Solís-Ortiz et al., 2010). Several studies have also focused on COMT because it is highly expressed throughout both the prefrontal cortex and the limbic system (Hong et al., 1998; Matsumoto et al., 2003), brain areas involved in cognition and emotion.

In humans, a number of studies have examined the influence of COMT on the electroencephalogram (EEG), a measure of brain function, because the dopaminergic system impacts cortical activity and reflects basic mechanisms of brain activation. The EEG is a recording of the rhythmical electrical activity of the brain that is thought to derive from extracellular current flow associated with summated postsynaptic potentials in synchronously activated, pyramidal cells that are perpendicular to the cortical surface (Mado and Zani, 2003; Miller, 2007). EEG measures the electrical cortical activity in the brain, with high temporal resolution, provides a direct measure of the present functional state of the brain and of its different levels of arousal (Niedermeyer, 1993a; Buzsáki, 2006). Resting EEG contains abundant information predictive of performance on several cognitive tasks (Basar and Güntekin, 2008; Solís-Ortiz et al., 2012) and has been useful for the diagnosis of neurodegenerative diseases (Fonseca et al., 2011). Moreover, the EEG technique enables the analysis of the effects of genetic polymorphisms on human brain function (Hodgkinson et al., 2010).

Human studies using EEG markers have demonstrated that COMT polymorphisms can affect the resting-state EEG global power, even though there are only a few studies reporting this in the literature. In young healthy men, Val allele carriers showed lower power than Met allele carriers within the upper *α* range, which was associated with executive performance in wakefulness (Bodenmann et al., 2009). In healthy subjects of both genders, the Val allele was associated with increased EEG delta/theta activity at Pz-Fz and increased extraversion scores (Wacker and Gatt, 2010). In women, the Met/Met genotype was associated with higher anxiety scores and low-voltage alpha resting EEG (Enoch et al., 2003). Other studies have found enhanced error positivity in individuals with the Met/Met genotype compared with Val carriers (Frank et al., 2007), low functional connectivity in healthy young women with the Met/Met genotype (Lee et al., 2011) and lower baseline prefrontal activation in individuals with the Val/Val genotype (Gianotti et al., 2012).

The effect of the COMT genotype on EEG activity in postmenopausal healthy women is not well known. One feature of women over 50 years is a decrease in serum levels of estrogens that produces significant physiological effects (Santoro and Tortoriello, 1999). There is an influence on dopaminergic function in striatum (Becker, 2000). Estrogen functions as a multipurpose brain messenger that can interact with neurotransmitter systems at critical brain nuclei and facilitate neuronal function via gene expression and transmitter-gated ion channels (Ostlund et al., 2003). It has been reported that estrogen is a regulator of COMT promoter activity. There are two estrogen response elements in the COMT promoter and that estrogen at physiological concentrations inhibits COMT mRNA expression in cells expressing estrogen receptors (Xie et al., 1999). The estrogen-mediated decrease in COMT mRNA is accompanied by a decrease in COMT activity (Jiang et al., 2003). This inhibitory regulation by estrogens is consistent with evidence that women with high estrogen states have higher COMT activity than other women with low levels of estrogens (Briggs and Briggs, 1973). Compared with men, women have higher striatal [^18^F] fluorodopa uptake, suggestive of greater presynaptic DA synthesis (Laakso et al., 2002), a lower D2 receptor affinity that reflects higher DA levels (Pohjalainen et al., 1998) and greater DA transporter uptake (Mozley et al., 2001). However, estrogenic state (menopausal or menstrual cycle) has not been fully taken into account in EEG studies of COMT activity and may be a significant confounder (Harrison and Tunbridge, 2008). DA mechanisms may be particularly relevant in prefrontal functions in women with low levels of estrogen in the middle age (Solís-Ortiz et al., 2010).

Because of the different regional expression patterns of COMT in postmortem human brains, the Val^158^Met polymorphism is thought to modulate dopaminergic neurotransmission most prominently in the prefrontal cortex, (Akil et al., 2003; Chen et al., 2004). Whether the differential expression of COMT modulates resting-state EEG regional power in postmenopausal healthy women remains unexplored. The aim of the current study was to examine the effects of the COMT Val^158^Met genotype on EEG activity in the frontal region, which is associated with executive functions such as self-control, planning, reasoning, abstract thinking and working memory (Baddeley and Logie, 1999). This study also seeks to determine whether COMT genotype also impacts EEG activity in the parietal region, an area associated with attention, sensory, verbal and visuospatial processes, and in the midline region, which is involved in attention, cognitive and emotional processing (Pizzagalli et al., 2006). This study hypothesized that the resting-state EEG power spectra in the frontal, parietal and midline regions during the eyes-closed and eyes-open states would be different depending on the COMT genotype in postmenopausal healthy women.

## Materials and Methods

### Participants

A total of 74 womens responded to recruitment advertisements. All respondents were genotyped for the Val^158^Met single COMT polymorphism. Of the 74 respondents, 24 healthy postmenopausal female volunteers between 48 and 65 years old with intact uteruses were selected based on their genotypes, 13 homozygous Val/Val allele carriers and 11 homozygous Met/Met allele carriers. The sample size of 24 womens with the Val^158^Met polymorphism was calculated to yield an expected power of 0.91 to detect a difference of 10% on EEG spectral bands with a two-sided significance level of *α* = 0.05. Since COMT enzyme activity is downregulated by estrogen (Jiang et al., 2003) and seems to affect the prefrontal cortex (Schendzielorz et al., 2011), only postmenopausal women were selected for this study. All women were given a medical history interview to assess their health status. To participate in the study, women must have been amenorrheic for at least 12 months and have no history of cardiovascular, metabolic, endocrine or malignant diseases. None of the participants were taking any type of medication at the time of the study and none had ever received hormonal treatment. The participants did not report a history of severe sleep disturbances. Participants were tested in a single session (between 09:00 h and 10:00 h) by one trained female. Participants were instructed to abstain from caffeine, alcohol and smoking and to sleep for 8 h on the day prior to testing. This study was approved by the Ethics Committee of the Department of Medical Sciences at the University of Guanajuato for Research on Human Subjects and is in accordance with the Declaration of Helsinki. All subjects provided written informed consent prior to participating in the study.

### Genotyping

Genomic DNA was extracted from peripheral blood leukocytes using standard methods. The portion of exon 4 that contains the polymorphic site was amplified by PCR. The total reaction volume was 27 μl and contained 25 pmol of forward (5′-TACTGTGGCTACTCAGCTGTGC-3′) and reverse (5′-GTGAACGTGGTGTGAACACC -3′) primers, 100 ng of genomic DNA, 2 mM MgCl_2_, and 250 μM dNTPs. The PCR conditions were as follows: denaturation at 94°C for 1 min, followed by 30 cycles of denaturation (94°C, 30 s), annealing (56°C, 30 s), and extension (72°C, 30 s), and a final extension cycle at 72°C for 10 min. The PCR products were digested at 37°C overnight with Hsp92ll (Promega), electrophoresed in a 4% agarose gel, and stained with ethidium bromide. There were two expected digestion products: Val/Val homozygote (114 bp), and Met/Met homozygote (96 bp) (Worda et al., 2003).

### EEG Recordings

All EEG records began at 09:00 h and finished at 10:00 h during rest, with the subjects’ eyes open and closed. During the recordings, participants were instructed to relax comfortably in a chair and place their chin on an individually adjusted head-rest. The recording session consisted of a 3 min period with eyes closed and a 3 min period with eyes open. EEG responses were recorded with 32 tin electrodes using an electrode cap (Compumedics Neuroscan Quick-Cap™, Charlote, NC, USA). The electrodes were positioned according to the International 10–20 system. Eye movements were recorded by an electrode 1 cm lateral to the left eye. Electrode impedance was less than 10 kΩ. EEGs were recorded on a 40 channel Scan model digital amplifier (NuAmps, Neuroscan, Charlote, NC, USA), set to pass frequencies from 0.5 to 35 Hz and gain 19. EEG activity was recorded on a personal computer at a sampling rate of 512 Hz and was analyzed off-line with SCAN 4.3 data analysis software (Compumedics Neuroscan, Charlote, NC, USA). EEG activity was carefully inspected for eye movement artifacts using the Artifact Rejection command integrated into the Edit Module, with appropriate parameters. Artifact rejection (eye movements, blinks, muscular activation, or movements artifacts) was performed on the raw EEG trace by posing a market at the onset of the artifact signal and a further market at the end of the artifact. The artifact segments (the EEG signal between the two markers) were excluded from the analysis. The free-epochs average of each subject from the resting-state EEG (Möcks and Gasser, 1984) was Fast Fourier transformed (FFT). Using traditional definitions of bands for characterizing EEG frequency spectra of normal EEG of the waking adult (Niedermeyer, 1993b; Davidson et al., 2000), the absolute power (Hz) was obtained for the following bands: delta, 0.5–4.0 Hz; theta, 4.0–8.0 Hz; alpha1, 8.0–11.0 Hz; alpha2, 11.0–14.0 Hz; beta1, 14.0–25.0 Hz; and beta2, 25.0–35.0 Hz. All bands of resting EEG power in the frontal (F3, F4, F7, F8, FC3, FC4), parietal (CP3, CP4, P3, P4) and midline (Fz, FCz, Cz, CPz, Pz and Oz) cortical regions, were analyzed to determine whether genotype-dependent differences between Val/Val and Met/Met carriers with eyes open and closed vary with scalp location. At least 100 s of EEG recording were analyzed for each condition (eyes open and eyes closed) in the all participants. The average time analyzed was 124 ± 30 s.

### Statistical Analysis

Statistical analyses were performed with STATISTICA for Windows 8 (StatSoft, Inc). The statistical power of the sample was estimated using the Sample Size Calculation and power analysis module in STATISTICA. We used *α* (two-tailed) = 0.05 and *β* = 0.20 to detect a standardized effects size of 10% between the two groups (Altman, 1991).

Before statistical procedures were applied, the data were tested for a normal distribution using Levene’s test. A Mann-Whitney *U*-test was used to compare the demographic characteristics between the Val/Val and Met/Met COMT genotypes, due to the non-normal distribution of these variables. To examine group differences in resting state EEG absolute power, and differences in the eyes-closed and eyes-open conditions, we conducted a repeated measures analysis of variance. The factorial ANOVA 2 × 3 repeated measures designs included 2 between-subjects factors (Genotype: Val/Val genotype and Met/Met genotype) and 3 within-subjects factors (Region: frontal, parietal, midline; Bands: delta, theta, alpha1, alpha2, beta1, beta2; Condition: eyes open, eyes closed). Significant interactions were analyzed using separate ANOVAs and follow-up contrasts were computed to explain the nature of significant effects. Age was included as a covariate in all analyzes. We computed *η*^2^ values in each ANOVA as a measure of effect size. Statistical analyses included a general linear model. Bonferroni corrected *post hoc* comparisons were computed to assess which genotypes differed from each other with the significance level set at *p* < 0.05.

## Results

### Characteristics of Participants

COMT genotype did not deviate from Hardy-Weinberg equilibrium (*X*^2^ = 0.946). The characteristics of the participants are shown in Table 1. The Val/Val and Met/Met individuals did not differ significantly education level, age at menarche, menopausal years, systolic arterial tension, diastolic arterial tension, height, body mass index, and pregnancies. Age tended to be higher in the Met/Met group. However, age did not produce significant differences when it was included as a covariate in all analyzes.

**Table 1:** **Participant characteristics**.

	Val/Val (*N* = 13)	Met/Met (*N* = 11)	*U*	p*
Age (years)	55 (48–61)	59 (51–65)	39.0	0.06
Years of education	3 (1–5)	3 (1–5)	62.0	0.58
Menarche (years)	13 (11–16)	13 (11–14)	69.0	0.88
Menopause (years)	43 (31–51)	47 (35–57)	46.5	0.15
Partus	2 (0–5)	4 (0–17)	53.5	0.30
Height (cm)	1.58 (1.53–1.64)	1.51 (1.12–1.77)	39.5	0.06
TAS (mmHg)	107 (90–140)	115 (90–140)	43.5	0.10
TAD (mmHg)	79 (60–90)	74 (50–90)	49.0	0.19
BMI (Kg/m2)	26.20 (20.58–33.28)	29.13 (20.78–38.44)	46.0	0.14

Values are shown as the median and range. *Mann-Whitney U Test. BMI = Body Mass Index. TAS = Systolic arterial tension. TAD = Diastolic arterial tension.

### Genotype Effects on Resting State EEG

We conducted an analysis of the absolute power derived from EEG data in participants with Val/Val and Met/Met genotypes for eyes-closed and eyes open rest conditions. Age did not show a significant influence on the EEG analysis. The omnibus analysis revealed main effects of Genotype (*F*_(1)_ = 7.16, *p* = 0.008), Band (*F*_(5)_ = 541.24, *p* = 0.000001), Region (*F*_(2)_ = 7.41, *p* = 0.0007) and Condition (*F*_(1)_ = 38.25, *p* = 0.000001), indicating that individuals with Val/Val and Met/Met genotypes differed in the absolute power of their EEG and that the differences varied by band, region and condition.

The differences were also seen in a Genotype -by-Band interaction (*F*_(5)_ = 3.30, *p* = 0.005), Band-by-Region interaction (*F*_(10)_ = 4.43, *p* = 0.000004), Condition-by-Band interaction (*F*_(5)_ = 13.97, *p* = 0.000001), Condition-by-Region interaction (*F*_(2)_ = 6.93, *p* = 0.001) and Condition-by-Band-by-Region interaction (*F*_(10)_ = 2.84, *p* = 0.002). Given the main effects of Genotype, Band, Region and Condition, and several interactions we conducted ANOVAs and analysis of contrasts to elucidate the group effects (Val/Val/ and Met/Met) across regions, bands and conditions. Follow-up analysis showed that in individuals with the Met/Met genotype, delta activity was increased in the eyes-closed condition (*F* = 4.95, *p* = 0.03, *η*^2^ = 0.66, *p* = 0.11 corrected), theta activity was increased in the eyes-closed (*F* = 7.02, *p* = 0.008, *η*^2^ = 0.18, *p* = 0.03 corrected) and eyes-open conditions (*F* = 8.67, *p* = 0.003, *η*^2^ = 0.24, *p* = 0.007 corrected), and the alpha1 band (*F* = 9.67, *p* = 0.002, *η*^2^ = 0.25, *p* = 0.004 corrected), alpha2 band (*F* = 19.14, *p* = 0.00002, *η*^2^ = 0.50, *p* = 0.00003 corrected), and beta1 band (*F* = 5.40, *p* = 0.02, *η*^2^ = 0.54, *p* = 0.04 corrected) were increased in the eyes-closed condition (Table 2).

**Table 2:** **Results of factorial ANOVA of absolute power between Val/Val and Met/Met genotypes with eyes closed and open**.

		COMT Val^158^Met genotype	
		Val/Val	Met/Met
Condition	Band	mean (SD)	mean (SD)	*F*	*p*	Effect size, *η*^2^	Bonferroni correction
Eyes-closed	Delta	14.62 (0.76)	17.12 (0.83)	4.95	**0.03**	0.66	0.11
	Theta	4.33 (0.18)	5.04 (0.20)	7.02	**0.008**	0.18	**0.03**
	Alpha 1	7.01 (0.53)	6.92 (0.58)	0.01	0.91	0.0007	3.63
	Alpha 2	4.43 (0.38)	4.83 (0.41)	0.52	0.47	0.02	1.88
	Beta 1	1.42 (0.06)	1.59 (0.07)	3.38	0.07	0.28	0.27
	Beta 2	0.40 (0.04)	0.46 (0.04)	0.87	0.35	0.30	1.40
Eyes-open	Delta	14.00 (1.14)	16.18 (1.24)	1.66	0.20	0.74	0.40
	Theta	3.50 (0.17)	4.24 (0.18)	8.67	**0.003**	0.24	**0.007**
	Alpha 1	2.59 (0.13)	3.18 (0.14)	9.67	**0.002**	0.25	**0.004**
	Alpha 2	1.96 (0.11)	2.67 (0.12)	19.14	**0.00002**	0.50	**0.00003**
	Beta 1	1.15 (0.08)	1.42 (0.09)	5.40	**0.02**	0.54	**0.04**
	Beta 2	0.45 (0.06)	0.48 (0.07)	0.12	0.73	0.007	1.46

### Interactions

**Figure 1:**
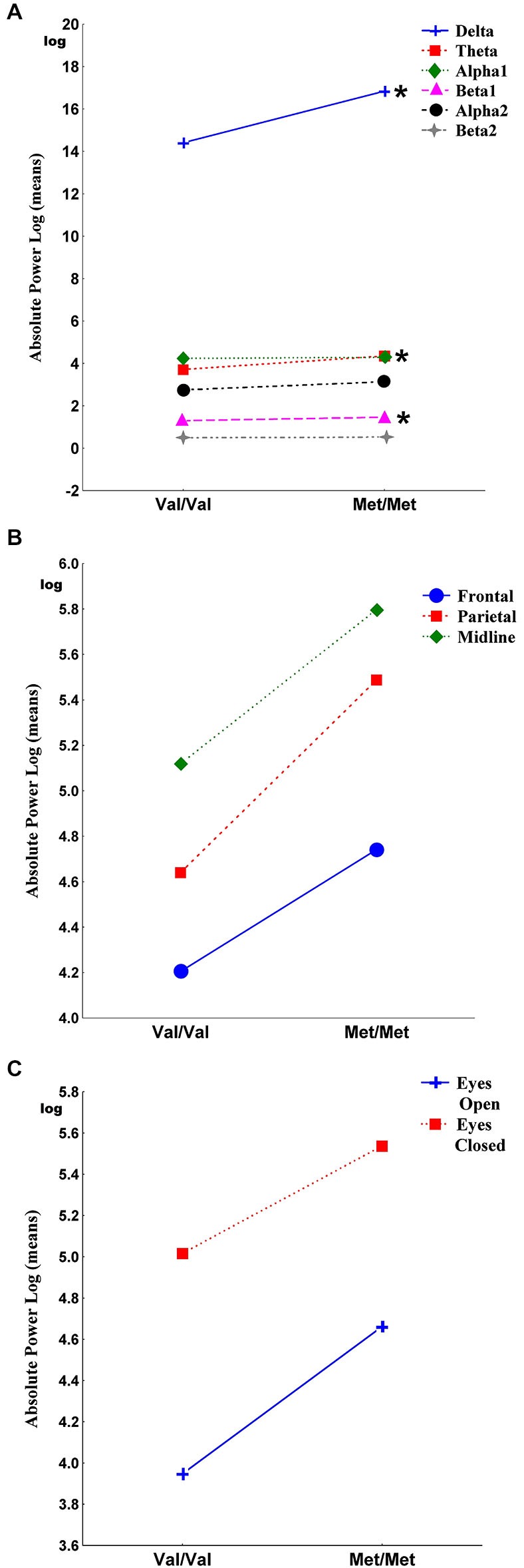
**It shows the main effects of the interactions of genotypes for band, region and condition**. Panel **(A)** shows the Genotype-by-Band interaction (*p* = 0.006) of delta, theta, alpha1, alpha2, beta1 and beta2 absolute power, log transformed (derivations pooled). Panel **(B)** shows the Genotype-by-Region interaction (*p* = 0.87) of frontal, parietal and midline regions (regions pooled). Panel **(C)** shows the Genotype-by-Condition interaction (*p* = 0.77) of eyes open and eyes closed (conditions pooled). Asterisks indicate the significance between Val/Val and Met/Met genotypes (*p < 0.05).

Figure 1 shows the main effects of the interactions of genotypes for band, region and condition. The Genotype-by-Band interaction was significant (*F*_(5)_ = 3.30, *p* = 0.006) (Figure 1A). Univariate results revealed that Met homozygote individuals exhibited more delta (*F* = 4.66, *p* = 0.03), more theta (*F* = 13.94, *p* = 0.0002) and more beta1 (*F* = 9.41, *p* = 0.002) than individuals with the Val/Val genotype whereas the alpha1, alpha2 and beta2 bands were not significantly different between genotypes. The Genotype-by-Region interaction was not significant (*F*_(2)_ = 0.14, *p* = 0.87) (Figure 1B), indicating that EEG power in the frontal, parietal and midline regions was not affected by interactions between genotypes. The Genotype-by-Condition interaction was not significant (*F*_(1)_ = 0.09, *p* = 0.77) (Figure 1C), indicating that EEG power in the eyes open and eyes closed conditions was not affected by interactions between genotypes.

## Discussion

The results of this study demonstrate the effects of the COMT Val^158^Met polymorphism on resting-state EEG power during the eyes-closed and eyes-open states in healthy women. The main finding was a significant interaction between genotypes and EEG spectral bands.

The analysis of this interaction revealed that Met homozygote women exhibited more delta, more theta and more beta1 activity than women with the Val/Val genotype, indicating a significant effect of genotypes. Our follow-up analysis showed that women carriers of the Met/Met genotype, who had more DA available at the synapse, exhibited augmented absolute power in the slow components of the spectrum: delta increased in the eyes-closed condition, theta increased in both eyes-closed and eyes-open conditions, and alpha1 increased in the eyes open conditions. The absolute power of the fast frequencies alpha2 and beta1were also increased in the eyes-open conditions than women carriers of the Met/Met genotype. Interestingly, we did not detect a Genotype by Region interaction, indicating that resting-state EEG power in the frontal, parietal and midline regions was not affected by interactions between genotypes.

One explanation for the lack of COMT effects on EEG regional power could be that the study lacked sensibility or statistical power to detect an effect. The number of participants included in our study was 24, which exceeded the number required for a sample size with statistical power (*n* = 3) according to our analysis. Indeed, in the contrast analysis, a significant effect was found for beta1 activity, which was elevated in the parietal and midline regions in individuals with the Met/Met genotype. Furthermore, the beta1 band showed a medium effect size in the sample, which describes the ratio of variance explained in the dependent variable. However, most of the comparisons between EEG regional power and genotypes did not survive correction for multiple comparisons, even after restriction of the analyses to certain regions and conditions. Our results are partially consistent with a study that investigated the effect of the COMT genotypes on BOLD activation during a working memory task conducted with male and female healthy subjects (Stokes et al., 2011). These authors did not find that COMT genotype effected the prefrontal cortical modulation of activity. Others studies that analyzed connectivity between the hemispheres found low functional connectivity in healthy young women with the Met/Met genotype (Lee et al., 2011) and decreased prefrontal connectivity in the frontal regions in individuals with Val/Val genotype (Liu et al., 2010).

It is important to note that the women analyzed in the present study were healthy volunteers without any apparent history of hyperactivity disorder, anxiety, depression or psychiatric disorders, which might influence EEG activity. COMT genotype has been shown to affect resting state EEG regional power in psychiatric patients. One study found that Met/Met patients with schizophrenia exhibited lower alpha activity at frontal and temporal sites than Val/Val patients (Venables et al., 2009). Another study found that schizophrenic subjects with the Met/Met genotype showed smaller P300 amplitudes than subjects with the Val/Val genotype in the frontal region during an auditory oddball paradigm (Gallinat et al., 2003). Low-voltage alpha in resting EEG was associated with higher anxiety scores in women with Met/Met genotype (Enoch et al., 2003). In a heterogeneous sample including both genders, increased resting posterior-versus- frontal delta/theta EEG activity was associated with increased extraversion scores in Val allele carriers (Lee et al., 2011). These reports suggest that significant changes in EEG activity related to COMT genotype are observed in individuals with mental disorders.

The resting EEG is a dynamic index of cortical activation and is therefore an intermediate phenotype for many behaviors in which arousal is implicated. Although COMT may not have a direct effect on resting-state EEG regional power, we found evidence that COMT genotype effects resting-state EEG spectral power. Our findings of higher absolute power in the delta, theta and alpha1 bands in individuals with the Met/Met genotype suggest a state of lower activation, whereas the increase of alpha2 and beta1 absolute power suggests a state of higher activation compared to women carriers of the Val/Val genotype. Although it is not possible in the present paradigm to draw conclusions about the consequences of resting-state EEG power on the behavioral, emotional and cognitive changes reported in the COMT genotypes, some interesting correlations may be considered and deserve further investigation. It has been reported that high absolute power of slow bands is related to cortical inhibitory processes (Steriade et al., 1990). Elevated theta and alpha1 power has been found in Met allele carriers with schizophrenia (Venables et al., 2009) and in individuals with attention-deficit/hyperactivity disorder (Barry et al., 2003) compared with healthy subjects. Brain activation studies have reported that the Met allele is associated with attenuated brain activation in the posterior cingulate gyrus and precuneus during the performance of an emotional task (Swart et al., 2011). High power of fast activity has been associated with arousal (Steriade, 1993), anxiety (Lee and Prescott, 2014), emotional reactivity (Tumyalis and Aftanas, 2014) and depression in females (Jaworska et al., 2012), suggesting increased emotive arousal. Although we did not measure the affective state of the participants, we observed alpha2 and beta1 absolute power increased in the individuals with the Met/Met genotype compared to women carriers of the Val/Val genotype, which may reflect arousal. Healthy individuals with the Met/Met genotype experienced larger subjective stress responses (Hernaus et al., 2013) and higher scores in the disorganization domain of the SPQ-B personality inventory (Sheldrick et al., 2008). Future studies on the COMT gene and EEG activity should consider the emotional status of the participants.

The EEG profile of the COMT Val^158^Met polymorphism described in our study may be related to the resting state functional brain through dopamanergic effects. Our findings are supported by the tonic and phasic DA theory and the influence of COMT on dopamanergic activity (Bilder et al., 2004). This theory assumes that COMT serves to modulate the balance of tonic and phasic DA function subcortically, and overall DA transmission cortically, the effect of the Met allele would be to increase tonic DA levels and decrease phasic DA release in subcortical regions, and increased DA concentrations in cortex, thereby enhancing the functions associated with tonic DA system activity and D1 stimulation cortically. In contrast, the Val allele would be expected to have complementary effects, increasing phasic DA transmission and D2 stimulation subcortically, while decreasing tonic DA neurotransmission subcortically and decreasing overall DA concentrations in the prefrontal cortex, thereby reducing cortical D1 neurotransmission.

These effects of the COMT gene may have an impact on the resting-state EEG spectral power of postmenopausal healthy females.

In conclusion, we have shown that the COMT Val^158^Met polymorphism does not directly impact resting-state EEG regional power modulation. Instead, we found that individuals with the Met/Met genotype showed a state of lower activation in resting-state EEG spectral power, denoted by delta and theta activities, and a state of higher activation denoted by beta1 activity, which may have functional effects. These results imply that COMT genotype status impacts resting-state EEG spectral power. These EEG profiles may serve as an endophenotypes, reflecting cortical influence of the COMT genotype on tonic and phasic dopamanergic function in healthy females.

## Author Contributions

SS conceived and designed the study, performed the experiments and data analysis, and drafted the manuscript. EP and MG carried out the molecular genetics analysis. MG performed the experiments and data analysis. All authors have read and approved the final manuscript.

## Conflict of Interest Statement

The authors declare that the research was conducted in the absence of any commercial or financial relationships that could be construed as a potential conflict of interest.
